# Supplementary figures and images for: Ancestral Chromatin Configuration Constrains Chromatin Evolution on Differentiating Sex Chromosomes in Drosophila
Source: PLoS Genet. 2015 Jun 26;11(6):e1005331. doi: 10.1371/journal.pgen.1005331 (PMC4482674; doi:10.1371/journal.pgen.1005331)

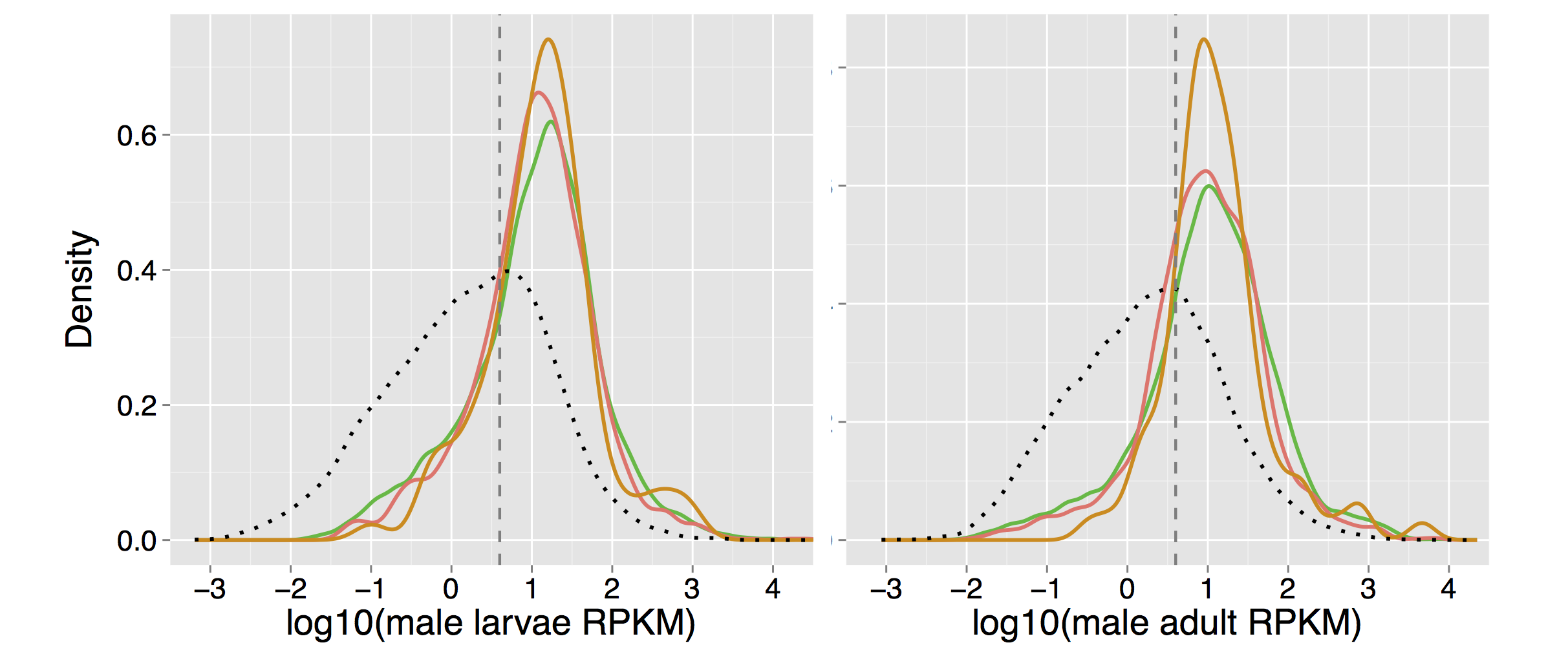

Supplement: S1 Fig — Shown are density plots of gene expression for protein coding genes on autosomes (green), the X chromosome (red) and dot chromosome (orange) of D. busckii male larvae and adults. We also plot the expression level of intergenic regions (dotted line), to determine a cutoff value (dashed line) for defining actively transcribed genes. (TIFF) [file pgen.1005331.s001.tiff]

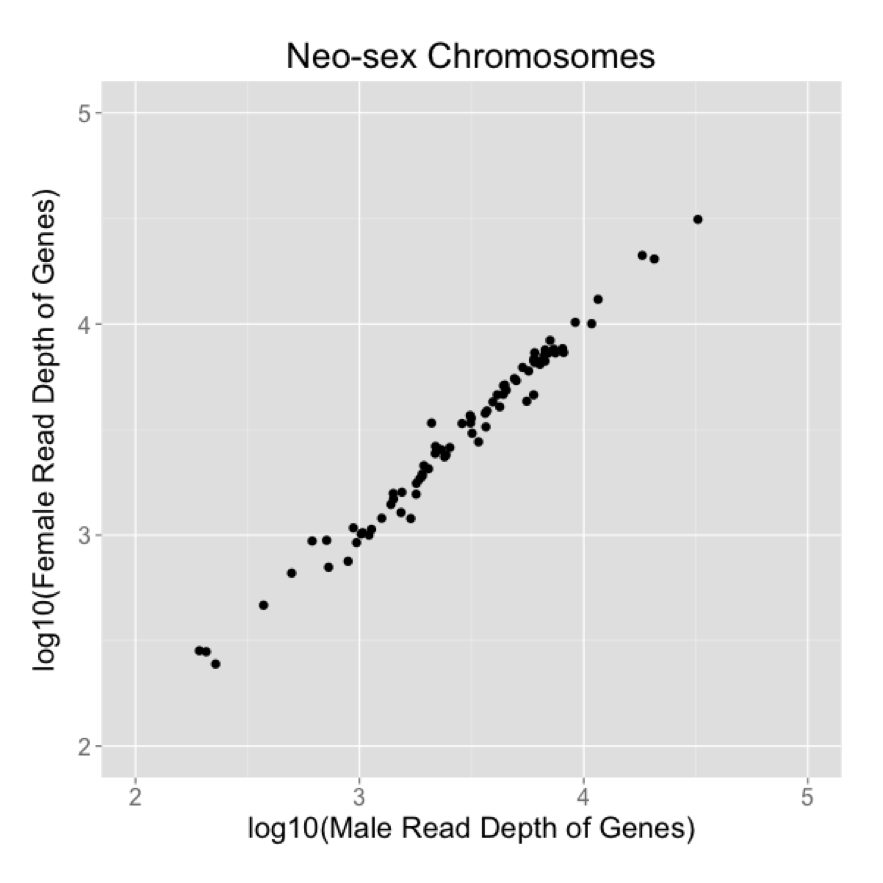

Supplement: S2 Fig — Shown is log10 based read coverage of neo-sex chromosome genes in males (x-axis) and females (y-axis). A similar level of coverage between sexes indicates that none of the neo-Y genes are deleted. (TIFF) [file pgen.1005331.s002.tiff]

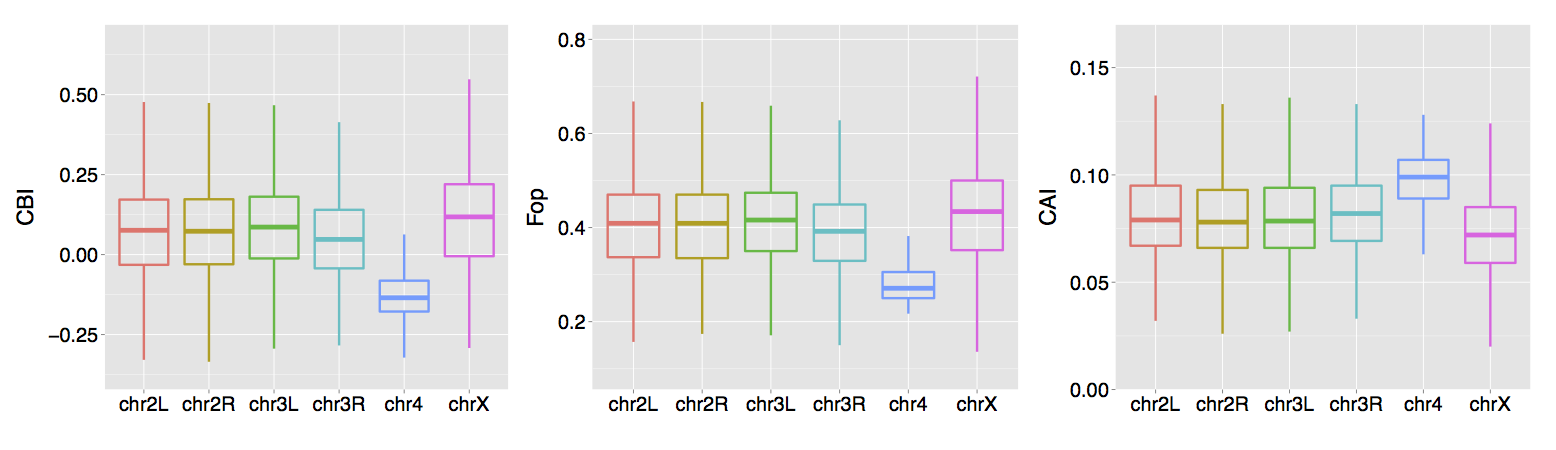

Supplement: S3 Fig — We compare levels of codon usage bias between genes on different chromosomes, using the neo-X sequences for the dot chromosome. Different measurements of codon usage bias, including codon bias index (CBI), frequency of optimal codons (as defined by D. melanogaster, Fop) and codon adaptation index (CAI) consistently show that dot-linked genes have reduced levels of codon usage bias. (TIFF) [file pgen.1005331.s003.tiff]

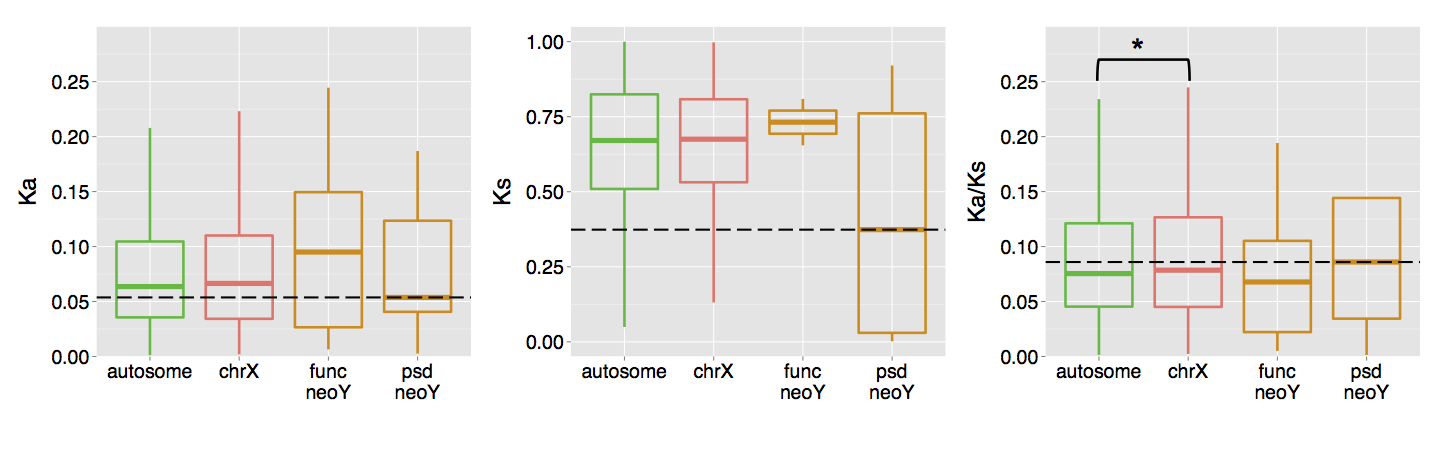

Supplement: S4 Fig — Shown are boxplots for K a, K s and the K a/K s ratio, for genes linked to autosomes, the X chromosome, and ancestral K a, K s and K a/K s ratios before neo-sex divergence for putatively functional neo-Y linked genes (func) and non-functional neo-Y linked genes (psd). We show Wilcoxon test significance level: P < 0.05: *, P<0.01: **, P<0.001: ***. (TIFF) [file pgen.1005331.s004.tiff]

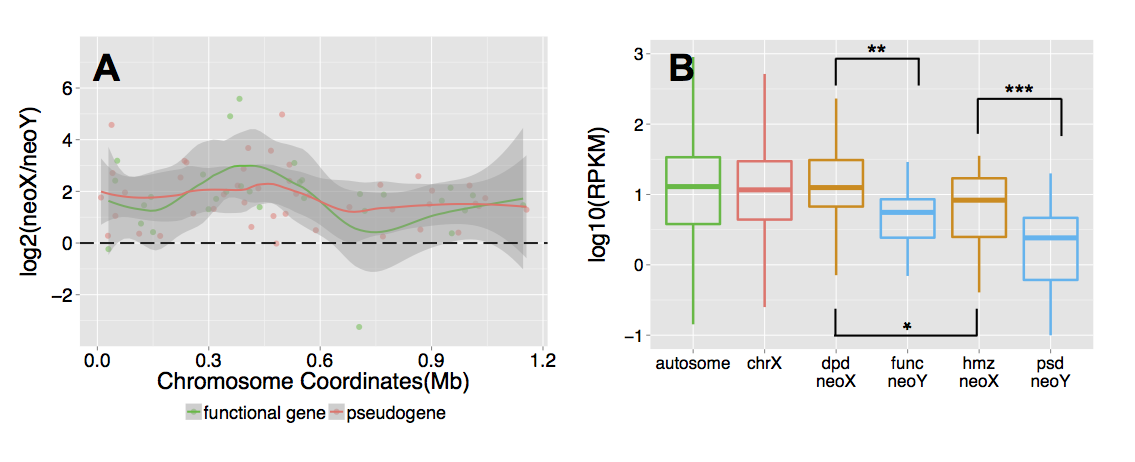

Supplement: S5 Fig — A. Shown is the relative male larvae expression of neo-X vs. neo-Y along the neo-sex chromosomes, with functional genes in green and pseudogenes in red. B. Boxplots of gene expression level of different chromosomes, with neo-sex linked genes divided into functional (func) and non-functional (psd) neo-Y genes, and their corresponding neo-X homologs (diploid vs. hemizygous neo-Xs, dpd vs. hmz). We show Wilcoxon test significance level: P < 0.05: *, P<0.01: **, P<0.001: ***. (TIFF) [file pgen.1005331.s005.tiff]

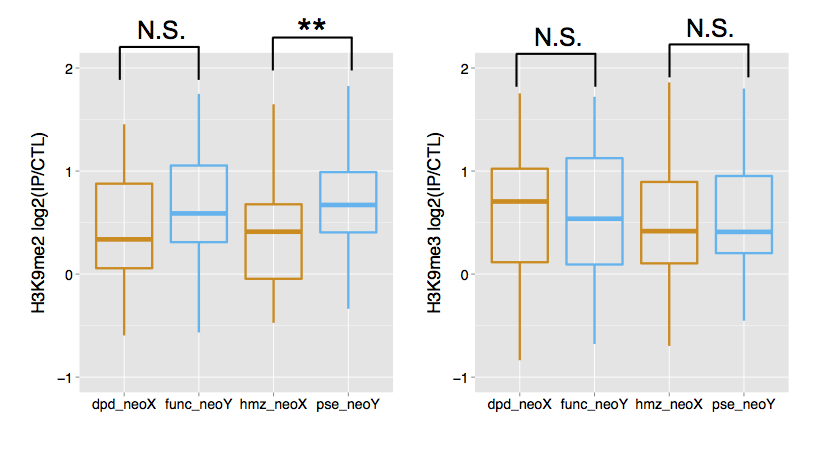

Supplement: S6 Fig — Boxplots showing the H3K9me2/3 enrichment level of functional and nonfunctional neo-Y linked genes (in blue), and their corresponding neo-X homologs. H3K9me2 but not H3K9me3 is significantly (Wilcoxon test, P<0.05) enriched on the non-functional neo-Y genes relative to their neo-X homologs. (TIFF) [file pgen.1005331.s006.tiff]

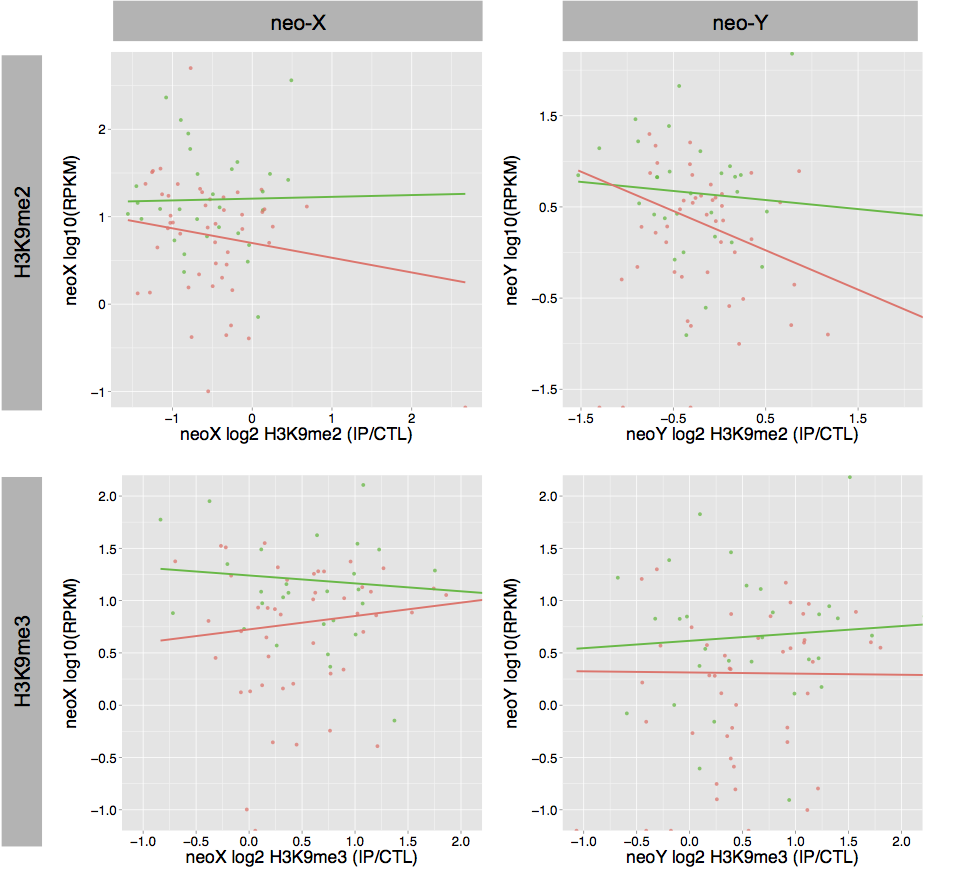

Supplement: S7 Fig — Shown are the normalized enrichment levels of H3K9me2 and H3K9me3 for neo-sex linked genes vs. their allelic gene expression level. Functional neo-Y genes and their neo-X homologs are in green, and non-functional neo-Y genes and their neo-X homologs in red. Only H3K9me2 shows a significant negative correlation (F-statistic test, P<0.05) with gene expression level on the neo-Y. Note that non-functional neo-Y genes show a stronger negative correlation between H3K9me2 enrichment and expression level than functional neo-Y genes. (TIFF) [file pgen.1005331.s007.tiff]

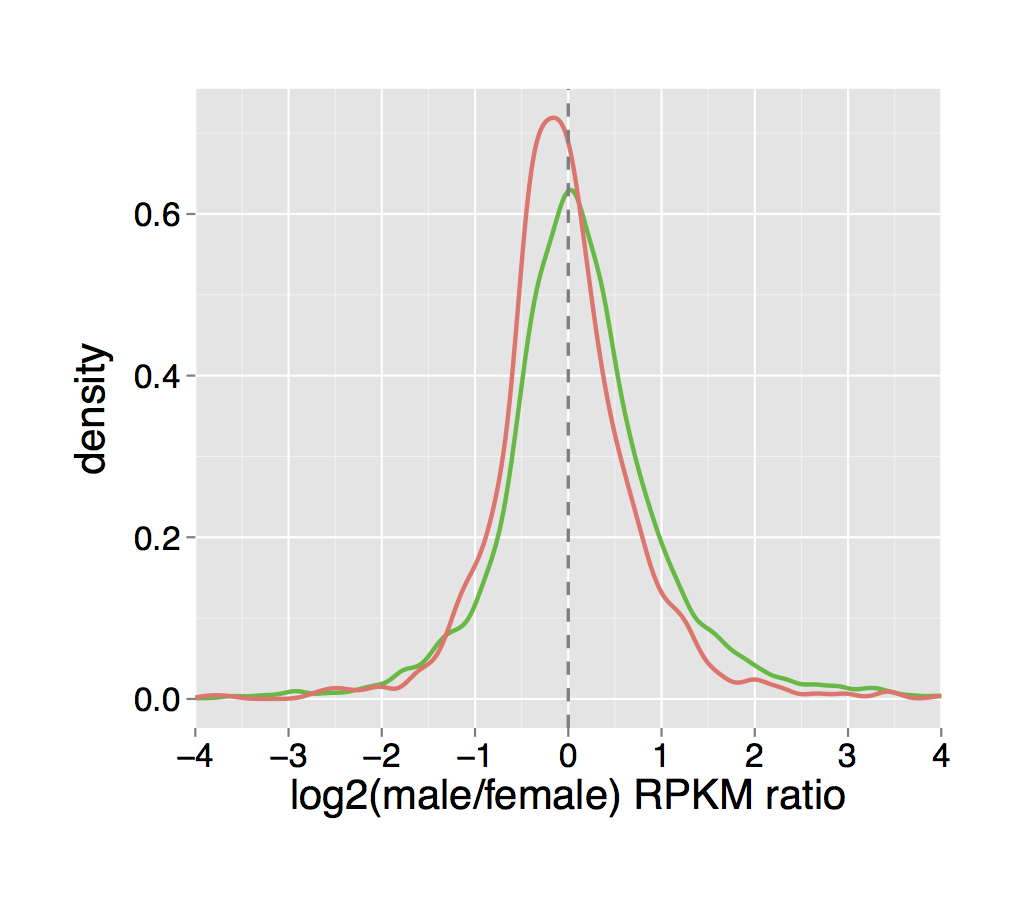

Supplement: S8 Fig — Density plot of male vs. female adult gene expression ratio, with X-linked genes in red, and autosomal genes in green. Most genes show equal expression levels between sexes, resulting in a peak centered at 0. Due to the demasculinization of X-linked genes (S1 Fig), this peak is shifted from 0 toward a lower relative expression in males. (TIFF) [file pgen.1005331.s008.tiff]

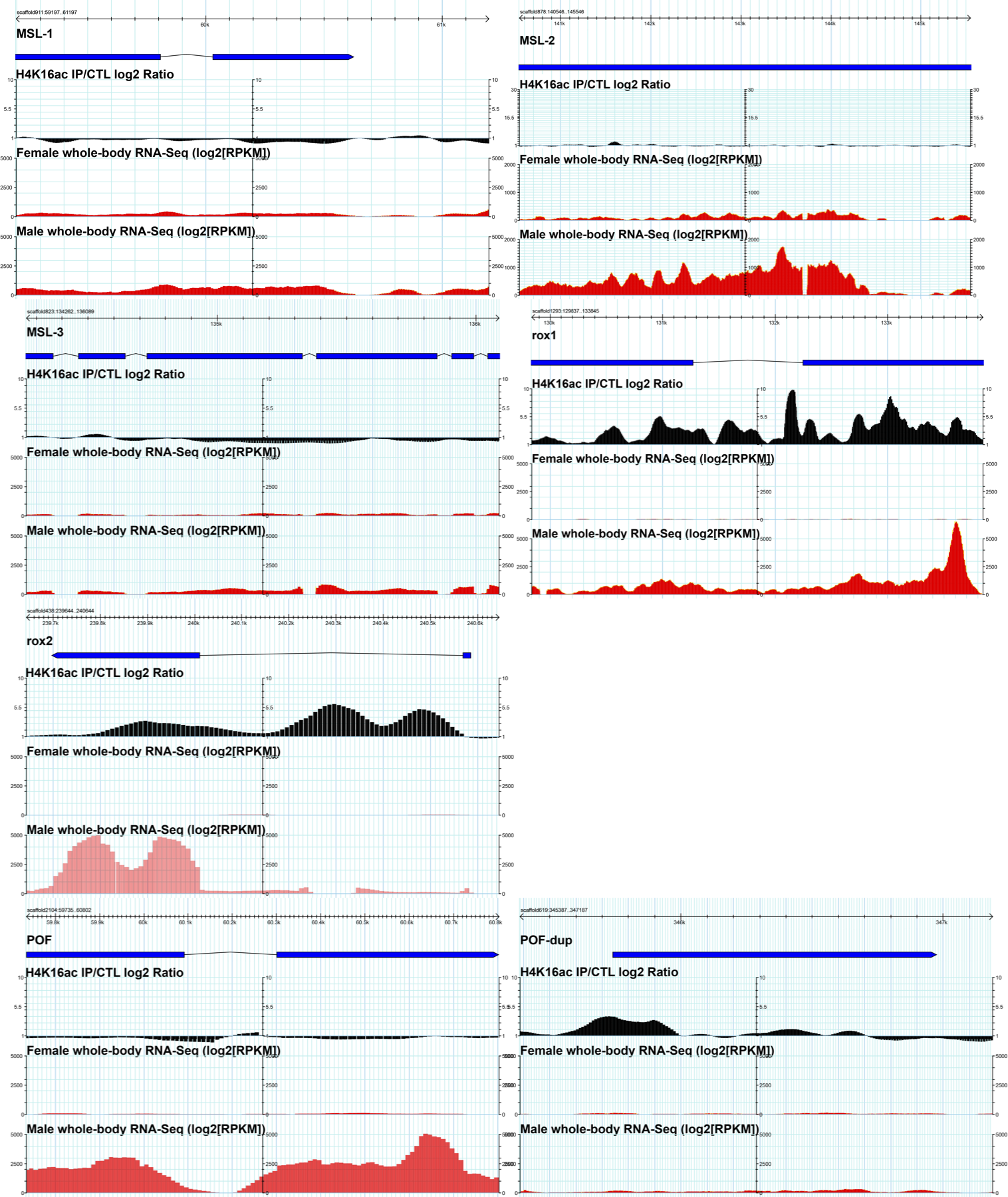

Supplement: S9 Fig — Shown are Gbrowser plots of MSL complex proteins, roX non-coding RNAs, and POF protein and POF duplicate protein of D. busckii. Their sex-biased gene expression pattern is consistent with their D. melanogaster orthologs. (TIFF) [file pgen.1005331.s009.tiff]

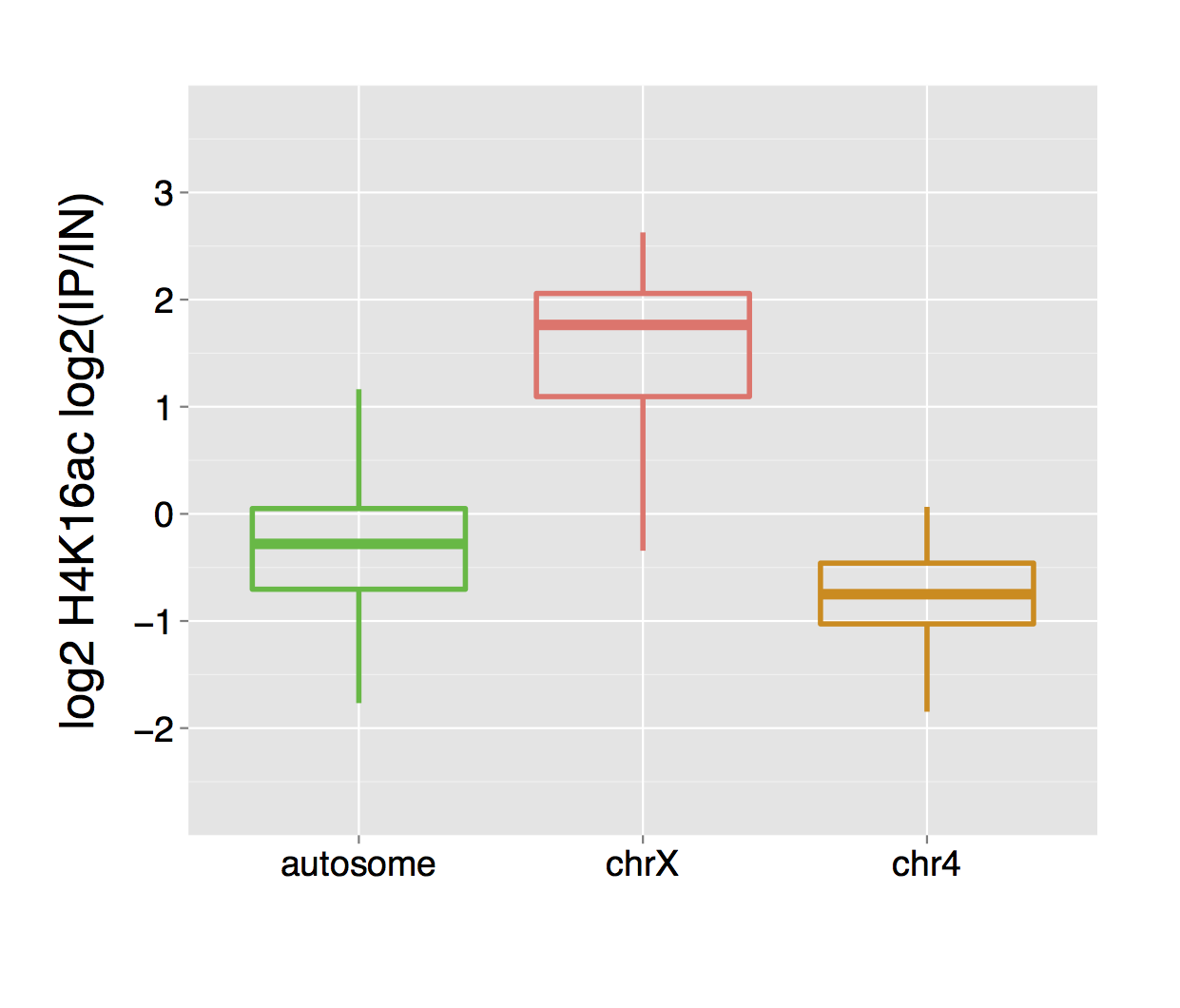

Supplement: S10 Fig — Shown are boxplots of log2 normalized H4K16ac enrichment levels from salivary glands of third instar male D. melanogaster larvae [66]. Note that the dot chromosome is deficient for the active H4K16ac mark. (TIFF) [file pgen.1005331.s010.tiff]

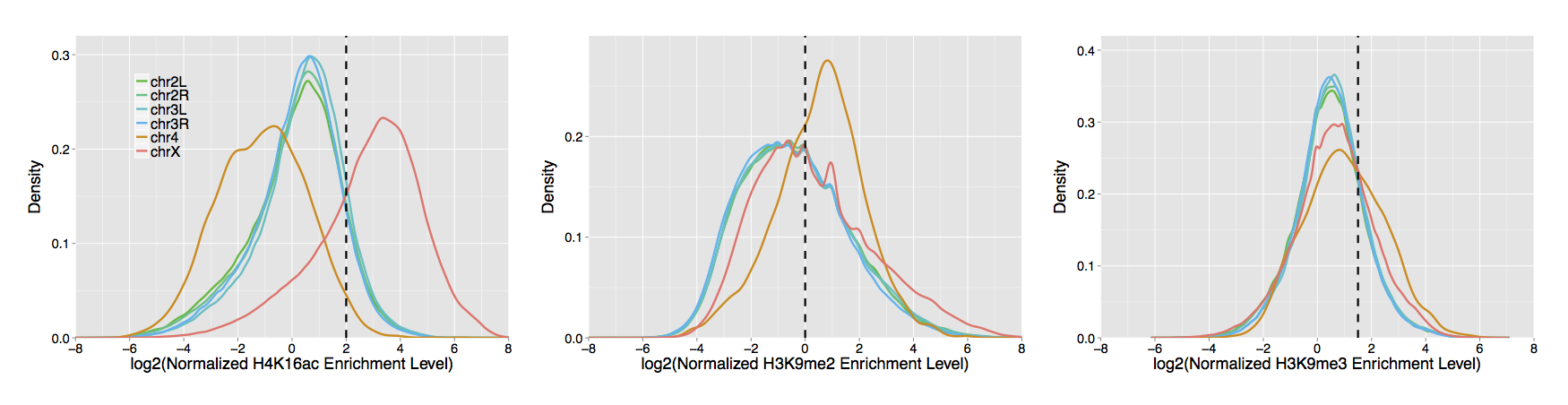

Supplement: S11 Fig — Shown are histograms of log2 normalized enrichment level of different chromatin marks within scaled bins of genes on different chromosomes from third instar larvae of male D. busckii. We determine an arbitrary cutoff (the dashed line) to define ‘bound’ or ‘unbound’ genes for a certain mark, which separates the distribution of sex or the dot chromosome from others. The chromosomes are named after their homologous D. melanogaster chromosomes. (TIFF) [file pgen.1005331.s011.tiff]
