# Supplementary material for: Ancestral Chromatin Configuration Constrains Chromatin Evolution on Differentiating Sex Chromosomes in Drosophila
Source: PLoS Genet. 2015 Jun 26;11(6):e1005331. doi: 10.1371/journal.pgen.1005331 (PMC4482674; doi:10.1371/journal.pgen.1005331)
Supplement: S1 Table — (DOCX) [file pgen.1005331.s012.docx]

**Table S1 Sequencing coverage of *D. busckii* genome**

| library insert size | number of read pairs | data collected (Gb) | coverage |
| --- | --- | --- | --- |
| female 170bp | 36955790 | 6.65 | 33.26 |
| female 365bp | 51696850 | 9.31 | 46.53 |
| female 451bp | 46530412 | 8.38 | 41.88 |
| female 5kb | 45570942 | 4.56 | 22.79 |
| female 10kb | 56974352 | 5.70 | 28.49 |
| male 500bp | 26773499 | 5.41 | 27.04 |
